# The Role of Carbohydrate Intake on the Gut Microbiome: A Weight of Evidence Systematic Review

**DOI:** 10.3390/microorganisms11071728

**Published:** 2023-06-30

**Authors:** Lorena P. Mora-Flores, Rubén Moreno-Terrazas Casildo, José Fuentes-Cabrera, Hugo Alexer Pérez-Vicente, Guillermo de Anda-Jáuregui, Elier Ekberg Neri-Torres

**Affiliations:** 1 Laboratorio de Biopolímeros, Departamento de Ingeniería Química, Industrial y de Alimentos—Universidad Iberoamericana Ciudad de México, Ciudad de México 01219, Mexico; p34390@correo.uia.mx; 2 Laboratorio de Microbiología, Departamento de Ingeniería Química, Industrial y de Alimentos—Universidad Iberoamericana Ciudad de México, Ciudad de México 01219, Mexico; ruben.morenot@gmail.com; 3 Departamento de Ingeniería Química, Industrial y de Alimentos—Universidad Iberoamericana Ciudad de México, Ciudad de México 01219, Mexico; jose.gustavo.fuentes@gmail.com (J.F.-C.); hugo.perez@ibero.mx (H.A.P.-V.); 4 Computational Genomics Division, National Institute of Genomic Medicine, Ciudad de México 14610, Mexico; 5 Center for Complexity Sciences, Universidad Nacional Autónoma de México, Ciudad de México 04510, Mexico; 6 Programa de Cátedras CONACYT, Consejo Nacional de Ciencia y Tecnología, Ciudad de México 03940, Mexico

**Keywords:** carbohydrates, gut microbiome, gut microbiota, food additives, weight of evidence

## Abstract

(1) Background: Carbohydrates are the most important source of nutritional energy for the human body. Carbohydrate digestion, metabolism, and their role in the gut microbiota modulation are the focus of multiple studies. The objective of this weight of evidence systematic review is to investigate the potential relationship between ingested carbohydrates and the gut microbiota composition at different taxonomic levels. (2) Methods: Weight of evidence and information value techniques were used to evaluate the relationship between dietary carbohydrates and the relative abundance of different bacterial taxa in the gut microbiota. (3) Results: The obtained results show that the types of carbohydrates that have a high information value are: soluble fiber with *Bacteroides* increase, insoluble fiber with *Bacteroides* and *Actinobacteria* increase, and *Firmicutes* decrease. Oligosaccharides with *Lactobacillus* increase and *Enterococcus* decrease. Gelatinized starches with *Prevotella* increase. Starches and resistant starches with *Blautia* decrease and *Firmicutes* increase. (4) Conclusions: This work provides, for the first time, an integrative review of the subject by using statistical techniques that have not been previously employed in microbiota reviews.

## 1. Introduction

### 1.1. Carbohydrates

**Figure 1:**
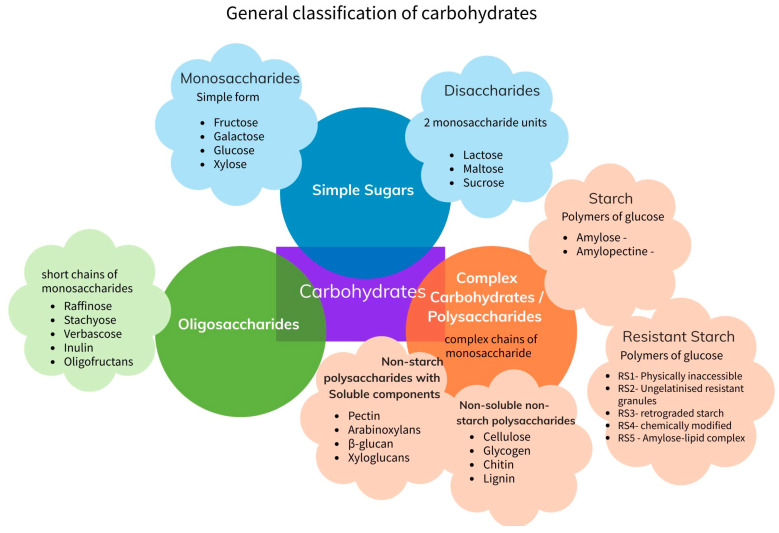
General classification of carbohydrates.

Carbohydrates (CH) serve as the main source of energy for humans, since these substances are easier to break down and convert to energy compared to other macronutrients [1]. Their general formula is Cn(H_2_O)_n_; their physicochemical, chemical, and biological properties are determined by their molecular arrangement. For instance, cellulose and starch are both glucose polymers, but their different molecular structures result in completely different properties. This review uses the Toussaint et al. (2009) [2] and Leong et al. [1] classifications: CHs as sugars, oligosaccharides, and polysaccharides. The Sajilata et al. [3] classification for starches is also included (see Figure 1).

Sugars are simple carbohydrates and are absorbed within the first minutes after ingestion. Monosaccharides and disaccharides are types of simple CHs that are composed of one or two molecules of saccharides. The most common examples of monosaccharides are glucose and fructose, while the most common examples of disaccharides are sucrose and lactose [4].

These compounds are commonly found in fruits and are easily absorbed in the small intestine as an immediate source of energy. Disaccharides are typically hydrolyzed by enzymes present in the small intestine, but if these enzymes are not available, the compounds will reach the large intestine and be degraded by the gut microbiota [4].

Oligosaccharides, such as inulin and oligofructans, are found in vegetable sources and are not easily digestible by human enzymes in the small intestine. Instead, they reach the large intestine undigested, where their hydrolysis depends on the available glycoside hydrolases (GHs) produced by the gut microbiota. Therefore, many oligosaccharides are not digested by humans and are instead fermented by the gut microbiota [5].

Soluble fibers such as pectin, which is a water-soluble polysaccharide found in the cell walls of vegetables, are partially hydrolyzed by a human enzyme in the small intestine, but an important portion reaches the colon [6], similar to the beta-glucan, non-starch polysaccharides, found in some cereals such as barley, oats, and wheat [7]. Arabinoxylans and xyloglucans are complex carbohydrates also found in vegetables; these also reach the large intestine undigested [8].

Starches are one of the most prevalent examples of carbohydrates. Not all starches are easily digested by humans. According to Sajilata (2006) [3]., starches can be classified according to their enzymatic hydrolysis:

- Rapidly digestible starches, which are hydrolyzed after less than 20 min of enzymatic digestion.
- Slowly digestible starches, which are absorbed in the small intestine and hydrolyzed in vitro after 100 min of enzymatic digestion.
- Resistant starches (RS), which are not hydrolyzed after 120 min of enzymatic incubation [3]. RS can occur naturally, such as potato starch [9], but also as a result of food processing (cooking techniques and production process). There are five RS types, depending on why these molecules resist hydrolysis [1]: type 1 RS is physically inaccessible because it is matrixed in food; type 2 RS is not gelatinized, and it is also inaccessible to enzymes; type 3 RS is retrograded after temperature changes, such as heating and cooling on multiple occasions; type 4 RS is a chemically modified starch where new bonds that are not α 1–4 or α 1–6 are present; and type 5 RS is amylose-lipid complex [10].

Finally, non-starch polysaccharides, such as chitin, might not be digestible for humans or their gut microbiome, and may exit the digestive tract intact [11].

#### 1.1.1. Carbohydrates as Part of Food

Historically, high-carbohydrate foods (cereals, tubers, legumes, fruits, and vegetables) and other carbohydrates found in mushrooms (*macromycetes*), fungi (*micromycetes),* algae, and foods of animal origin, have been part of the human diet across regions. Vegetables, tubers, legumes, and mushrooms have a high concentration of carbohydrates, but humans can absorb a limited amount of these. In some cases, these carbohydrate sources might be part of the traditional culinary culture or even traditional medicine [12].

In recent years, carbohydrates’ effect on the gut microbiota and the host’s health has gained global attention among scientists. There is a growing and particular interest in studying the carbohydrates found in widespread food sources, such as cereals, tubers, and legumes, that are processed in different ways.

#### 1.1.2. Carbohydrates as Functional Components; Functional Carbohydrates

As a result of the advancements in food processing technologies and the evolution of traditional medicine, nowadays, certain CHs are isolated and incorporated into food as functional ingredients [13].

Various vegetable and fungal species have been considered for their potential healing properties and have become part of traditional medicine among different cultures. In some cases, these substances are also added to food to obtain health benefits.

For the purposes of this review, these carbohydrates will be referred to as functional carbohydrates. In recent years, scientific interest in these substances has increased, leading to the discovery that, in some cases, these substances might be generating changes in the gut environment and influencing the host’s health.

Some of the functional carbohydrates that have been studied as gut microbiota modulators are: *Hylocereus undatus* [14], *Artemisia ordosica* [15], *Cyclocarya paliurus* [16], *Enteromorpha Clatharata* [17], and chondroitin sulfate, a polysaccharide of animal origin, that is found commonly in the connective tissue of animals [18].

#### 1.1.3. Carbohydrates as Food Additives

A wide range of carbohydrates are commonly used as food additives in order to modify the physical properties of processed foods, such as viscosity, texture, gelling, emulsifying, stabilizing, and crystallization control, among others. These long-chain polymer carbohydrates are classified as hydrocolloids [19].

These hydrocolloid carbohydrates have been evaluated in the search for toxicological effects, are approved by international entities, and their acceptable daily intake (ADI) has been established [20,21]. Some examples are guar gum [22], taro starch [23], pectin [24], potato starch [25], and transglycosylated starch [26].

Based on observations that strongly suggest the gut microbiota is an essential determinant of the host’s health, some studies have focused on the effect of carbohydrates that bypass digestion in the small intestine and enter the large intestine undigested, where they serve as substrates for the gut microbiota.

### 1.2. Gut Microbiota and Microbiome

The microbiota refers to a community of microorganisms that inhabit a specific environment [27]. Particularly, those residing in the human gastrointestinal tract constitute the human gut microbiota.

There are many definitions of the microbiome but, for the purposes of this document, Lederberg and McCray’s definition [28] will be used, which describes the microbiome as an “Ecological community of commensal, symbiotic and pathogenic microorganisms within a body space or other environment”. Another definition worth mentioning describes the microbiome as “a characteristic microbial community occupying a reasonable well-defined habitat which has distinct physicochemical properties” [29].

In particular, the human gut microbiota or human gut microbiome is estimated to have approximately 150 times more genes than the whole human genome and contains about 10^14^ microorganisms belonging to different species [30].

#### 1.2.1. Gut Microbiota General Composition

Bacteria is the most extensively studied domain of all the microorganisms found in the human gut. It has been shown that the presence of some phyla, families, genera, and species of bacteria has been associated with a reduced or increased risk of developing certain diseases, and the overall health state [31,32,33,34,35,36,37].

The main bacterial phyla found in the human gut are Firmicutes, Bacteroidetes, Actinobacteria, Proteobacteria, Fusobacteria, Cyanobacteria, Verrumicrobia, and Spirochaetes (Zeng et al., 2017); from those, the most abundant are Firmicutes and Bacteroidetes, representing more than 87% of the total gut microbiota [38,39,40,41,42].

#### 1.2.2. Factors That Modify the Microbiome 

**Figure 2:**
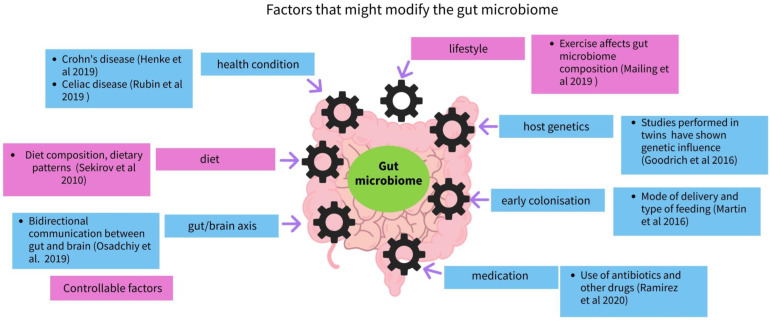
Factors that might modify the gut microbiome adaptation [31,43,44,49,50,51,52,53].

A wide range of factors influence the microbiota and the microbiome (see Figure 2) including the host’s genetics [43], early colonization [44], early feeding practices [45,46,47], living arrangements [48], having older siblings, [32] furry pets [44], the use of certain medications, especially antibiotics [49], and controllable factors such as diet and general lifestyle [50]. Among these controllable factors, diet, especially the carbohydrates contained in it, seems to have a significant effect on the modulation of the gut microbiome.

#### 1.2.3. Carbohydrates as Modulators of the Gut Microbiome 

Diet has an important impact on the gut microbiome; specific macronutrients, micronutrients, and bioactive compounds lead to changes in the composition of microbial communities in the gut.

As mentioned above, carbohydrate digestion requires GHs to hydrolyze these molecules. Humans produce only 17 GHs to break down carbohydrates in the upper gastrointestinal tract. At the same time, *Bacteroides thetaiotaomicron*, one of the most abundant bacterium in the gut, encodes, by itself, over 260 GHs, 15 times more than humans, allowing the human host to indirectly metabolize different carbohydrates [54,55], obtaining energy and different metabolites, such as short-chain fatty acids (SCFA) [16,56].

The presence and expressions of GH enzymes vary among bacterial species and the host’s gut environment. This relationship between ingested carbohydrates and the gut microbiome has been the subject of several studies.

To date, it has been difficult to compare results among studies that analyze the gut microbiota. On the one hand, this is due to differences in the experimental design (variations of the in vivo methodologies, the host species being studied, settings, type of intervention, sequencing techniques, and other potential confounding variables). On the other hand, there are differences in the bioinformatics and analysis tools (software used to analyze genomic sequences, databases consulted, and statistical methods used). For these purposes, the use of statistical tools that have been applied in other scientific fields, such as weight of evidence (WoE) and information value (IV), are being suggested in this work.

### 1.3. Statistics Weight of Evidence (WoE) and Information Value (IV)

Variable categorization and data binarization can facilitate the use of specialized algorithms to compare information from a wide range of topics, including studies that might differ in their methodologies.

Variable categorization is the process of dividing a set of variables into different categories based on their characteristics. This process is used in statistical analysis and data management in order to organize and better analyze data [57].

Data binarization is a processing technique that involves converting continuous or categorical variables into a variable that can only take two values. In some cases, this process involves creating different variables for each category of the original variable [58].

However, relying solely on these techniques may lead to overlooking certain patterns and repetitions in the data. To overcome this limitation, the use of WoE-IV is suggested. WoE is a transformation that uses the number of “bins” or events where the independent variable affects the response variable. IV is a technique obtained from the WoE that identifies important variables in a predictive model [59] (Fan and Ding 2022). These values can be calculated as follows [60]:

(1) $${WoE}_{i}=log\left(\frac{\frac{N_{i}}{\sum_{i=1}^{n}N_{i}}}{\frac{P_{i}}{\sum_{i=1}^{n}P_{i}}}\right)$$

(2) $$IV=\sum_{i=1}^{n}\left[\left(\frac{N_{i}}{\sum_{i=1}^{n}N_{i}}-\frac{P_{i}}{\sum_{i=1}^{n}P_{i}}\right)WoE_{i}\right]$$

The use of the WoE and IV have evolved from logistic methods and have been employed in various literature reviews to code variables related to different topics. The obtention of an IV has been applied to solve binary variable and binary classification problems in various fields such as credit scoring [61], marketing analytics [62], pharmacology [63], client conversion analysis, toxicology, and language processing tools. These techniques are useful for evaluating the IV of independent variables also referred to as predictive variables against potentially dependent variables, also referred to as response variables [61], allowing the selection of binary variables that can predict a specific outcome [62,63,64].

In recent years, this approach has been successfully applied to compare different clinical trials, even generating methodologies that unify the use of these techniques. The primary motivation for applying these statistical tools is to integrate statistically valid conclusions from numerous studies that were conducted with non-comparable methodologies or have variations in nature and experimental design [64]. Given the above, microbiome studies have the aforementioned limitations that cannot always be compared, as experimental designs vary widely.

The primary objective of this work is to examine the potential relationship between ingested carbohydrates’ characteristics including their origin, chemical composition, and other characteristics (predictive variables), and the changes in the relative abundance of microorganisms found in the gut microbiota at different taxonomic levels such as phyla, class, order, family, and genus (response variables).

This review uses the SPIDER tool [65,66], in addition to the statistical techniques of weight of evidence and information value, that have not previously been used in this type of research. These methods provide a structured approach to the design and conduct of systematic reviews, allowing for the identification of consistent patterns in the literature. This work aims to contribute to a better understanding of how different types of carbohydrates may modify the gut microbiota.

## 2. Materials and Methods

This review was carried out using the SPIDER tool [65,66], as shown in Table 1. The phenomenon of interest is the possible changes that might occur in the gut microbiome when oligosaccharides and polysaccharides, with different properties, functions, and origins, are consumed. The design of this review includes studies conducted in the past five years, written in English, involving healthy individuals (humans or laboratory animals) with a minimum intervention period of 14 days, and using molecular techniques to analyze the gut microbiota. The SPIDER tool was used to ensure a systematic approach to the review process.

For the study design (Table 1), articles published more than 5 years prior to the start of the review were excluded because the comparability of results could not be ensured, as sequencing techniques and databases may have changed significantly over time. Additionally, publications with intervention periods shorter than 14 days were also excluded to ensure sufficient time for variations in the gut microbiota to occur.

Articles involving the consumption of mono and disaccharides as the primary intervention were also excluded, as these carbohydrates are usually digested before they reach the large intestinal lumen. Furthermore, interventions using food additives with a non-carbohydrate molecular structure were excluded from the review. Only articles involving human or laboratory mammals, that could provide insights into the human gut microbiota, were included in order to provide information on human or human-like gut microbiota. Neonatal studies were excluded to compare only mature gut microbiomes.

Illness models without a healthy intervention group were also excluded, to avoid confounding variables. Review articles and models using in vitro techniques or for veterinary research were also excluded. For the purposes of this review, only articles with 16s gene shotgun sequencing or whole genome sequencing were included as an attempt to standardize the sequencing techniques.

### 2.1. Search Query

The study design resulting search query was: ((((“gut microbiome” [Title]) OR (“gut microbiota” [Title]) OR (faecal microbiota [Title]) OR (faecal microbiome [Title]) OR (fecal microbiota [Title]) OR (fecal microbiome [Title])) AND ((food additive [Title]) OR (dietary fiber [Title]) OR (polysaccharide [Title]) OR (polysaccharide [Title]) OR (oligosaccharide [Title]) OR (starch [Title]) OR (maltodextrin [Title])))) NOT (“in vitro” [Title]) NOT (review [Title]).

The research was performed on 9 March 2021 by using two different databases: Scopus and PubMed. These two databases were included as recommended by Siddaway, Wood, and Hedges [67]. A scientific landscape visualization was created using the VOSviewer software Mac version 1.6.16 [68,69] to analyze the abstracts of the resulting articles and to visualize the data.

### 2.2. Screening

The duplicated articles resulting from the search query were removed, and the remaining entries were screened to verify that the title and methodology match the interest and objective of the present review. The remaining studies were filtered according to the inclusion and exclusion criteria previously described.

### 2.3. Information Synthesis and Variable Categorization

The remaining articles were read thoroughly by the authors to understand the methodology, dietary intervention, carbohydrate source, and the results obtained from the intervention. The articles were labeled to categorize the information, according to the function or functions of the carbohydrate—food component, food additive, or functional carbohydrate—and were also classified according to the carbohydrate’s properties and chemical structure. Finally, the bacteria (phylum, order, family, genus, and species), whose relative abundance was significantly different from the control group, were also extracted by the authors. Subsequently, this information was categorized into binary variables to compare 47 studies, despite the methodological differences among them.

The information extraction, variable categorization, binarization, and statistical techniques used (WoE-IV) conform to the methodology proposed by the authors and are part of the contribution of this work.

For the purposes of this study, the variables were divided into two main classifications: predictive variables (independent variables) and response variables. The predictive variables were divided into two categories:

1. Carbohydrate use: food additive, functional carbohydrate, or food component
2. General description of the carbohydrate: Sulfated saccharide, fructan, inulin, oligosaccharide, polysaccharide, starch, gelatinized starch, resistant starch, insoluble fiber, soluble fiber, and antioxidant capacity.
  Within the response variables, there is only one classification:
3. Bacterial diversity variables (BDV): Bacterial taxa whose relative abundance showed a significant change compared to the control group after the intervention.

Independent variables were transformed into binary variables (binarized) where the presence of a use or characteristic was assigned a value of one “1” and the absence was assigned a value of zero “0”.

These variables were not defined as mutually exclusive because, for example, an intervention might contain a specific food that might be rich in starch but also in inulin. Another example would be that a specific carbohydrate might be used as a food additive but also be found as a natural food component.

Each response variable representing bacterial taxa was subdivided into two categories: the decrease and increase variable for each of the taxa based on the modification of their relative abundance compared to the control group. This allowed the creation of a table with only binary variables.

BDV (type 3) that showed a statistically significant difference in relative abundance in just one of the 47 articles were excluded due to insufficient data to conduct a statistical analysis on them. Bins (cooccurrence of a predictive and a response variable) that only appeared once during the process will not be deeply addressed in the discussion but will be mentioned in the results table.

### 2.4. Statistical Analysis and Co-Occurrence of Categorical Variables

Finally, a WoE-IV analysis was performed using each BDV (type 3) as the response variable and each type 1 and 2 variable (carbohydrate origin and description) as the predictive variable. IV scores were obtained for each pair (predictive vs. response), and those pairs with an IV score above 0.5, and at least two bins, will be further analyzed in the results and discussion section. The mathematical and statistical analysis was conducted using Python 3.10.4 [70] and implemented in Jupyter notebooks [71]. The Python libraries Pandas [72] and NumPy [73] AI tools were used for the grammatical editing of this article [74,75].

## 3. Results

### 3.1. Search Strategy

The search strategy yielded a total of 284 articles, the search on Scopus gave 255 hits, and the search on PubMed gave 150 hits; 121 articles were duplicated.

**Figure 3:**
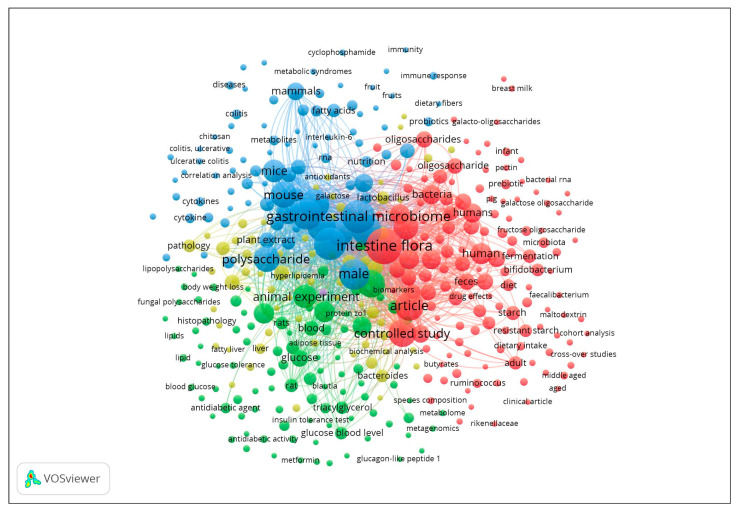
Research landscape universe made with VOS viewer.

The resulting scientific landscape model is shown in Figure 3. An interactive version of it can be consulted at: https://app.vosviewer.com/?json=https://drive.google.com/uc?id=1vGFc1sE_zyTH-uGcvguk1j3PrYp6Gpcq.

### 3.2. Screening 

**Figure 4:**
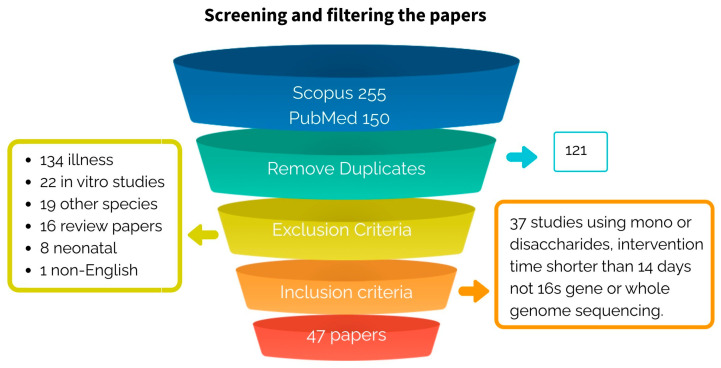
Screening and filtering the articles.

A total of 47 articles were included in the study; in other words, after removing duplicates from the two scientific databases, 16.5% of the 284 search query results were included (Figure 4). From the 284 articles: 47.2% were excluded because the study was performed on a specific illness or health problem without a healthy intervention group; 13% of the studies were excluded from the analysis because the experimentation was performed, either using traditional microbiological techniques to characterize the gut microbiota or had an intervention time of less than 14 days; 7.7% were performed in vitro; 6.7% were performed using a veterinary or marine approach; 5.6% of the publications were literature reviews; 2.8% were undertaken to neonate or very young human babies and; finally, one article was not written in English.

Some examples of studies that were also found in the search query but did not meet the inclusion criteria were:

- Studies without a healthy intervention group [76,77,78,79,80].
- In vitro studies [81,82,83].
- Studies carried out on neonates or very young infants [84,85].
- Studies conducted on other species [11,86,87].

Out of the 47 included articles: 36 were animal studies (76.5%) [14,15,16,17,18,23,24,25,26,88,89,90,91,92,93,94,95,96,97,98,99,100,101,102,103,104,105,106,107,108,109,110,111,112,113,114] and 11 human trials (23.4) [22,115,116,117,118,119,120,121,122,123,124]; 34 of them are on the topic of functional carbohydrates (72.4%); 31 are about carbohydrates naturally occurring in food (66%); and 12 address carbohydrates that can be used as food additives (25.5%). It is important to note that these three categories are not mutually exclusive.

A synthesis of the screened articles and the reasons for the exclusion are shown in Figure 4.

### 3.3. Information Synthesis and Variable Categorization 

For the construction of the categorical variable matrix, type 1 and 2 variables were extracted from the text and included as possible predictive variables (Table 2). As for the case of BDVs, type 3, a total of 74 bacterial variables were identified. However, 43 were removed due to their occurrence in only one publication, leaving 31 BDVs as possible response variables (Table 3).

### 3.4. Statistical Analysis and Co-Occurrence of Categorical Variables

As mentioned earlier, the number of bins for each possible combination of predictive and response variables was computed, followed by the calculation of the WoE and IV score for each pair of variables with at least one bin. Table 4 presents the number of bins and IV for variable pairs that achieved an IV score of at least 0.5.

## 4. Discussion

The trend of the studies herein included shows a scientific interest in identifying carbohydrates that might have a nutraceutical or medical application; in other words, functional carbohydrates—72.4% and considering carbohydrates naturally occurring in food—66% (food components). In addition, the proportion of articles where an experimental design was made using carbohydrates as food additives was 25.5%, indicating the interest in the impact of these ingredients on the gut microbiota. It is important to remark that “carbohydrate use” variables are not mutually exclusive; they can be used as food additives and used to improve the host’s health, as seen in guar gum and pectin carbohydrates, as mentioned in the Results section.

The analysis performed indicates that the structure of carbohydrates has an important IV on the gut microbiome composition, as shown in Table 4. Different starches, sulfated polysaccharides, fructans, and inulins have different IVs with specific BDVs.

Based on the studies included, it was observed that the most frequently reported bacterial taxa that showed changes during various dietetic interventions were the phylum *Firmicutes*, the genera *Bifidobacterium*, and *Lactobacillus*, each mentioned 12 times. High IVs were obtained only at the genus and phylum levels. For order, class, family, and species, the data obtained were insufficient to achieve any significant result.

The analysis showed that the phylum *Firmicutes* decreased in nine studies and increased in four studies. A decrease was observed with inulin and resistant starch in rats [110], pectin oligosaccharides in mice [24], purple sweet potato in mice [107], a fungal origin polysaccharide isolated from *Flammulina velutipes* in rats [100], a feruloylated oligosaccharide from maize bran in rats [111], wheat starch in rats [94], insoluble dietary fiber from pear pomace in rats [99], pigs fed with a diet rich in cellulose and xylose [108], and human intervention with dietary fiber [124]. An increase was observed in physically inaccessible resistant starch in mice [96], *Lycium barbarum* polysaccharide in mice [91], resistant starch in healthy human adults [118], and in diets enriched with wheat starch [94].

Regarding the genus *Lactobacillus*, which belongs to the *Firmicutes* phylum, two of the studies showed a decrease in this genre with the ingestion of *Artemisa ordosica* polysaccharide in rats [15] and *Sargassum fusiforme* polysaccharide in mice [88], while the rest of the studies in which this genus’ change was reported showed an increase in its relative abundance, following the ingestion of different carbohydrates. An increase was observed with the ingestion of pectin oligosaccharides in mice [24], bamboo-shaving polysaccharide in mice [105], *Lycium barbarum* polysaccharide in mice [91], mannan oligosaccharides in mice [95], oligosaccharides derived from dragon fruit *(Hylocereus undatus)* in mice [104], maize bran oligosaccharides in rats [111] galactooligosaccharides in mice [113], and chondroitin sulfate oligosaccharide in mice [18]. These observations will be further discussed according to the type of each polysaccharide.

The *Bifidobacterium* genus abundance increased by the ingestion of taro flour and starch in rats [23], pectin oligosaccharides in mice [24], mannan oligosaccharides in mice [95] resistant starch in human adults [117], and long-chain inulin in human aging adults [119].

Subsequently, the phylum *Firmicutes* and the phylum *Bacteroidetes* abundance were modified in 10 studies, 2 showing a decrease and 8 showing an increase. A decrease was observed in physically inaccessible resistant starch in mice [96] and resistant starch in human adults [118]. An increase was observed in inulin and rats fed with resistant starch [110], mice fed with pectin oligosaccharides (S. Zhang et al. 2019), mice fed with purple sweet potato oligosaccharide [107], rats supplemented with *Artemisia ordosica* polysaccharide [15], rats supplemented with a polysaccharide isolated from *Flammulina velutipes* [100], dietary fiber isolated from sweet potato residue in rats [103], gelatinized wheat starch included in mice diets [94], and dietary fiber from pear pomace administered to rats [99].

The following sections will address the significant changes observed in specific genera and phyla, based on the classification of the polysaccharides studied.

### 4.1. Sulfated Polysaccharides

Sulfated polysaccharides obtained a high IV (1.808, see Table 4) as a predictive variable for the reduction in *Lactobacillus.* These results are shown in Chen et al.’s study [130] using *Sargassum fusiforme* polysaccharide applied to mice [88] and Chen et al.’s fucoidan study in rats, where this phylum decreased significantly. Nevertheless, this trend differs from Gotteland et al.’s [131] review, which suggests that, by consuming sulfated polysaccharides, this phylum might increase. Sulfated polysaccharides also show a high IV (1.397, see Table 4) for predicting the increase in *Odoribacter*, as shown in Shang’s study [18] using chondroitin sulfate oligosaccharide. This IV is only sustained by one bin, and no further reference was found to sustain this IV so it will not be further discussed.

Sulfated carbohydrates also obtained a high IV (1.398, see Table 4), as a predictive variable for the increase in *Turicibacter*, as mentioned in a study by Zhu et al. [97], where mice were fed with sulfated carbohydrates from sea cucumbers and showed an increase in this genus. These bacteria are SCFA producers and have shown high heritability, according to Goodrich et al.’s study [43]. This genus showed a significant difference against the control group, twice in the 47 studies included.

It was also possible to observe a high IV (1.398, see Table 4) and only one bin when using sulfated polysaccharides as a predictive variable to increase the abundance of *Desulfovibrio* [18]. This gram-negative genus is sulfate-reducing to produce hydrogen sulfide [132] in the presence of sulfated carbohydrates. This bacterial taxon is considered to have a negative impact on the gut microbiome and the host’s health.

### 4.2. Gelatinized Starch

The second highest IV (1.781) and two bins (see Table 4) were obtained by evaluating gelatinized starch as a predictive variable for an increase in *Prevotella* abundance. The co-occurrence of this type of polysaccharide with the *Prevotella* genus was found in [23], where rats fed with taro (flour or starch) showed a significant increase in *Prevotella* compared to the control group, as well as in Pi et al.’s study [90], where pigs fed with corn starch showed a significant increase in the same genus. This trend was also consistent with Liu et al.’s in vitro study’s findings [103].

This genus is very interesting as part of the gut microbiome. On the one hand, it is related to plant-based diets, as mentioned by Martinez et al. [133], where different living conditions in the United States of America and Papua New Guinea are compared, and gut microbiota is associated with those environmental factors. Also, its relative abundance is higher in certain dietary patterns, such as the Mediterranean diet, as observed in De Filippis et al.’s studies [134]. This genus is reported to be a starch degrader and has been correlated in various studies with a beneficial impact on the immune system.

On the other hand, certain species and strains within the *Prevotella* genus have been associated with an increased risk of certain health conditions. For example, *P. copri* has been linked to intestinal inflammation and insulin resistance [135]. Is has also been implicated in rheumatoid arthritis [136] and shown to cause modifications that increase the inflammatory response [137]. Additionally, *P. copri*, along with other strains of the same genus, are more abundant in HIV-1 infected subjects [138]. These findings, coupled with the high genetic diversity of the genus, lead to the current definition of some *Prevotella* species and strains as possible pathobionts [135,136,137,138,139].

Gelatinized starch also obtained a high IV (1.398, see Table 4) as a predictive variable for the reduction in *Proteobacteria*, as indicated in Do et al.’s study [94], where diets containing gelatinized wheat starch were related to a decrease in *Proteobacteria*. A high abundance of this phylum has been linked to the host’s difficulty in maintaining a balanced gut microbiome, indicating that a high prevalence of *Proteobacteria* might indicate dysbiosis [140]. The role of *Proteobacteria* is discussed by Zhang et al. [141], Wang et al. [142], Everard et al. “a” [143], Everard, et al. “b” [144], Ridaura, et al. [145], Larsen et al. [139], and Xiuying Zhang et al. [146]. It is also important to mention that the *Escherichia coli* species belongs to the *Proteobacteria* phylum.

### 4.3. Fungal Polysaccharides

Mushroom and fungal polysaccharides also obtain a high IV (1.398, see Table 4) for the reduction in *Proteobacteria.* This phylum includes several pathogenic bacteria, suggesting that some of these polysaccharides might have a protective effect against dysbiosis [114,140], indirectly improving the host’s health.

### 4.4. Oligosaccharides

A high IV (1.051 and six bins, see Table 4) for oligosaccharides as predictive variables for an increase in *Lactobacillus* abundance was found; thus, the studies included have very different sources of oligosaccharides: using pectin oligosaccharides [24], mannan oligosaccharides [95], dragon fruit Hylocereus-undatus-derived oligosaccharides [104], feruloylated oligosaccharides from maize bran [111], galactooligosaccharides [113], and chondroitin sulfate oligosaccharide, all in murine models.

This genus consists of gram-positive bacteria, and some species have shown probiotic activity modifying the gut environment and improving the host’s health [18,147,148,149,150]. The IV and number of bins obtained also supports the idea that vegetable oligosaccharide sources might have a prebiotic function [151,152,153,154].

Oligosaccharides also have a high IV (0.947 and two bins, see Table 4) for predicting a decrease in *Enterococcus;* this genus is considered a commensal microorganism [24,104].

### 4.5. Insoluble Fiber

Insoluble fiber had a high IV (0.95 and two bins, see Table 4) for the increase in the phylum Actinobacteria in feruloylated oligosaccharides from maize bran [111] and alkali-soluble polysaccharides from *Arctium lappa* L. [89]. This phylum, along with the *Proteobacteria*, represents approximately 10% of the gut microbiota and has been shown to have a crucial role in the gut homeostasis [155]. It is also important to mention that the *Bifidobacterium* genus is part of this phylum. Insoluble fiber also had an IV (0.828 and 4 bins, see Table 4) when used as a predictive variable for *Bacteroides* increase, and an IV (0.754 and 5 bins, see Table 4) when used as a predictive variable for *Firmicutes* reduction. This result is important, as the number of bins is high, reinforcing the already known relationship between insoluble fiber and this genus.

### 4.6. Starch

A high IV (0.947 and 2 bins, see Table 4) has been found when using starch as a predictive variable for an increase in *Firmicutes*, possibly due to the physical inaccessibility of resistant starch. *Firmicutes* are gram-positive bacteria, some of which are also butyrate producers [96]; the IV is maintained when resistant starch is seen as the predictive variable, as in Kaur et al.’s [96] results. It is believed that an increase in *Firmicutes* leads, inevitably, to a decrease in the *Bacteroidetes’* relative abundance [118].

### 4.7. Soluble Fiber

Soluble fiber scored an IV (0.729 and 5 bins, see Table 4) for predicting an increase in *Bacteroides.* The number of bins obtained reinforces the positive association between this phylum and soluble fiber. This genus has a great capacity to degrade diverse carbohydrates [156]. Soluble fiber also scored an IV (0.500 and 2 bins, see Table 4) for predicting an increase in *Actinobacteria*. This phylum is important for maintaining the gut homeostasis [155].

### 4.8. Inulin

Inulin has a moderate IV (0.5375, and 3 bins, see Table 4) for predicting an increase in *Bifidobacterium*. This genre of microorganisms is considered probiotic; this effect was observed in aging individuals [119], in a clinical trial using inulin-type prebiotics [122], and in a human randomized crossover trial by using chicory inulin-type fructan contained in snack bars [115]. This bifidogenic effect has also been observed in multiple in vitro studies, such as those by the authors of references [154,157].

### 4.9. Carbohydrates as Food Additives and Natural Food Components

The variables used to define carbohydrate use—food additive, functional carbohydrate, and food component—do not seem to have a significant effect on the gut microbiome. Even though these variables appeared frequently during the review, they only had two high IVs; food additives for predicting *Blautia* decrease (0.842 and 2 bins, see Table 4). *Blautia* is a bacterial genus that has shown prebiotic effects but also correlates with certain diseases when it dominates the gut microbiota. More research about this genus and its specific species and strains needs to be carried out in order to understand these microorganisms as part of the gut microbiota [158].

The variable “carbohydrates naturally occurring in food” had a high IV for predicting *Bacteroides* increase (0.828 and 2 bins, see Table 4), matching the fact that soluble and insoluble fiber are common in different food sources. *Bacteroides* is a genus that metabolizes carbohydrates very efficiently and contains several species that have a probiotic effect [159].

### 4.10. Carbohydrates as Functional Compounds (Functional Carbohydrates)

A high IV was not observed for any BDV and functional carbohydrates; this might be because each one of these compounds interacts with the gut microbiome individually, modifying specific BDVs.

To summarize, some pairs of predictive and response variables have four or more bins, such as insoluble fiber with *Firmicutes* reduction, soluble and insoluble fiber with *Bacteroides* increase, and oligosaccharides with *Lactobacillus* increase. These relationships are already well-defined in microbiome studies. However, there are pairs of variables with high IVs and two to three bins that are not already well established that point to areas of opportunity for further research. For example: gelatinized starch and *Prevotella* increase; resistant starch and *Firmicutes* increase; sulfated polysaccharides with an increase in *Desulfovibrio*, *Turicibacter*, and *Odoribacter* and a decrease in *Lactobacillus*; gelatinized starch with a reduction in *Proteobacteria*; inulin with a rise in *Faecalibacterium*; starch and resistant starch with a reduction in *Blautia*; and inulin with an increase in *Bifidobacterium* (see Table 4).

This might point towards existing relationships that could become well-established by future research if the results obtained are consistent with those obtained in this work. It would be in the authors’ interest to monitor the publications on the topic continuously, to keep the database growing and find more robust results. It is also essential to mention that this review includes studies on both human and laboratory mammals. Although they are similar, their gut microbiota might not react in the same way in the presence of different carbohydrates. Authors should discuss the results and how they can be interpreted from the perspective of previous studies and of the working hypotheses. The findings and their implications should be discussed in the broadest context possible. Future research directions may also be highlighted.

## 5. Conclusions

The results obtained through the specific methodology used, for the first time, in this weight of evidence systematic review reinforce previous observations suggesting that differences in carbohydrate consumption have an important role in the host’s health by impacting the composition of the gut microbiota and the microbiome environment.

To begin with, the SPIDER tool was used to determine the specific focus of the review by defining the phenomenon of interest, inclusion and exclusion criteria, and research type in order to establish a proper framework for identifying the key variables and later binarizing them to enable the use of WoE.

The use of WoE as the primary statistical approach allowed the obtaining of results that align with well-known relationships between specific carbohydrates and their effect on BDVs in the gut microbiome, such as oligosaccharides and *Lactobacillus* increase. The fact that these relationships were observed and corroborated by this approach not only strengthens previous knowledge but also provides evidence that the entire methodology is effective. However, this might open new research interests for continuing to study and feed this model and, in this way, establish new carbohydrate–bacterial diversity relationships.

## Figures and Tables

**Table 1:** SPIDER tool description.

SPIDER Tool Search Description		
S	Sample	Fecal or gut samples from humans or animals that could provide insights into the human gut microbiome.
P I	Phenomenon of Interest	This study aims to investigate changes in the microbiome composition following dietary interventions with different oligosaccharides and polysaccharides, which can be found naturally in food, added as food additives, or be functional carbohydrates found in nature.
D	Design	Inclusion Criteria: Articles written in English published within the past five years. Studies involving preclinical (animal) or clinical (human models). Studies including groups with healthy individuals, excluding those where all the intervention groups had baseline diseases or diseases intentionally generated during intervention through genetic modification, drug administration, or diet. Experimental design with a minimum intervention period of 14 days. Studies using molecular techniques, such as 16s gene shotgun sequencing or whole genome sequencing. Exclusion Criteria: Neonatal studies. Articles involving the consumption of mono and disaccharides as a primary intervention. Articles involving the consumption of non-carbohydrate food additives as a primary intervention. Review articles and models using in vitro techniques, or for veterinary purposes. Articles that were carried out in illness models without a healthy intervention group.
E	Evaluation	Gut or fecal microbiome composition, as well as some biomarkers and/or histological changes.
R	Research type	Quantitative.

**Table 2:** Categorical variables used as possible predictives.

Type	Variable Name	Number of Appearances	References
1	Carbohydrates as food additive	12	[22,23,24,25,26,96,105,107,109,110,116,121]
1	Carbohydrates as functional components (Functional carbohydrates)	34	[14,15,16,17,18,22,23,24,25,88,89,91,93,95,97,98,100,101,102,103,104,106,107,110,111,112,113,115,116,117,118,119,122,125]
1	Carbohydrates naturally occurring in food (Food components)	31	[14,17,18,23,24,25,89,91,93,94,96,98,99,100,101,102,103,104,105,107,108,111,113,114,116,118,119,120,121,124,126]
2	Fungal polysaccharides	3	[100,102,114]
3	Polysaccharides with antioxidant capacity	10	[15,17,88,89,91,95,101,102,114,127]
3	Sulfated polysaccharides	3	[18,88,97]
3	Fructans	4	[104,115,119,122]
3	Inulin	4	[92,115,119,122]
3	Oligosaccharides	11	[14,18,24,95,98,104,106,109,111,113,122]
3	Polysaccharides	18	[15,16,17,88,89,91,93,97,100,101,102,105,107,108,112,114,116,120]
3	Starch	11	[23,25,26,90,94,96,116,117,118,120,121]
3	Gelatinized starch	3	[23,90,94]
3	Resistant starch	11	[25,26,90,96,110,116,117,118,120,121,123]
3	Insoluble fiber	11	[89,90,92,93,97,99,103,108,110,111,124]
3	Soluble fiber	16	[22,24,89,90,92,93,97,98,103,108,109,110,112,119,122,124]

**Table 3:** Table of type 3 bacterial diversity variables (BDVs) used as response variables.

Microorganism Taxa	Decrease	Increase
*Actinobacteria*	1 [101]	3 [22,111,128]
*Actinobacteria_Bifidobacterium*	n/a	13 [14,17,23,24,90,95,104,113,115,117,119,120,122]
*Bacteroidetes*	2 [96,118]	8 [15,24,94,99,100,103,107,110]
*Bacteroidetes odoribacter*	2 [18,99]	n/a
*Bacteroidetes_Bacteroidales_oscillospira*	n/a	2 [16,107]
*Bacteroidetes_Oscillospira_Ruminococcus*	n/a	4 [15,109,120,123]
*Bacteroidetes_Alistipes*	n/a	2 [15,99]
*Bacteroidetes _bacteroides*	n/a	7 [90,92,97,109,111,112,113]
*Bacteroidetes_prevotellaceae*	n/a	3 [18,91,123]
*Bacteroidetes_Prevotellaceae_prevotella*	n/a	4 [16,23,90,98]
*Firmicutes*	9 [24,94,99,100,107,108,110,111,124]	4 [91,94,96,118]
*Firmicutes_Enterococcus*	3 [24,97,104]	n/a
*Firmicutes_lactobacillus*	3 [14,15,88]	10 [17,18,24,91,95,104,105,111,112,113]
*Firmicutes_Clostridia*	n/a	2 [16,96]
*Firmicutes_Clostridium*	2 [111,113]	2 [116,124]
*Firmicutes_Lachnospiraceae_blautia*	3 [93,116,121]	n/a
*Firmicutes_Ruminococcaceae_Faecalibacterium*	n/a	2 [120,122]
*Firmicutes_Coprococcus*	2 [121,122]	n/a
*Firmicutes_Lachnospira*	2 [115,121]	n/a
*Firmicutes_Roseburia*	n/a	3 [15,97,120]
*Firmicutes_Turicibacter*	n/a	2 [25,97]
*Proteobacteria*	2 [94,114]	2 [91,108]
*Proteobacteria_Sutterella*	n/a	2 [23,25]
*Proteobacteria_Desulfovibrio*	n/a	2 [18,109]
*Tenericutes*	3 [25,101,122]	n/a
*Verrucomicrobia_Akkermansia*	n/a	3 [25,91,103]
*Verrucomicrobia _Akkermansia_muciniphila*	n/a	2 [17,123]

**Table 4:** Table of WoE-IV/Bins count.

Predictive Variable	Response Variable BDV	Information Value (IV)	Number of Bins	References
Sulfated polysaccharides	*Lactibacillus* reduction	1.808	1	[88]
Gelatinized starch	*Prevotella* increase	1.781	2	[23,90]
Fructan	*Faecalibacterium* increase	1.435	1	[122]
Sulfated polysaccharides	*Desulfovibrio* increase	1.398	1	[18]
Fungal polysaccharides	*Proteobacteria* decrease	1.398	1	[114]
Gelatinized starch	*Proteobacteria* decrease	1.398	1	[94]
Sulfated polysaccharides	*Turicibacter* increase	1.398	1	[97]
Sulfated polysaccharides	*Odoribacter* increase	1.397	1	[18]
Fructan	*Coprococcus* increase	1.144	1	[122]
Inulin	*Faecalibacterium* increase	1.144	1	[122]
Oligosaccharides	*Lactobacillus* increase	1.051	6	[18,24,95,104,111,113]
Insoluble fiber	*Actinobacteria* increase	0.95	2	[111,129]
Starch	*Blautia* decrease	0.948	2	[24,116]
Resistant starch	*Blautia* decrease	0.948	2	[24,116]
Oligosaccharides	*Enterococcus* reduction	0.947	2	[24,104]
Resistant Starch	*Firmicutes* increase	0.947	2	[96,118]
Starch	*Firmicutes* increase	0.947	2	[96,118]
Carbohydrates used as food additives	*Blautia* decrease	0.842	2	[116,121]
Carbohydrates naturally occurring in food	*Bacteroides* increase	0.828	2	[111,113]
Insoluble fiber	*Bacteroides* increase	0.828	4	[90,92,97,111]
Insoluble fiber	*Firmicutes* reduction	0.754	5	[99,108,110,111,124]
Soluble fiber	*Bacteroides* increase	0.729	5	[90,92,97,109,112]
Sulfated polysaccharides	*Roseburia* increase	0.677	1	[97]
Sulfated polysaccharides	*Enterococcus* reduction	0.676	1	[97]
Inulin	*Bifidobacterium* increase	0.538	3	[115,119,122]
Fructans	*Enterococcus* reduction	0.509	1	[104]
Soluble fiber	*Actinobacteria* increase	0.500	2	[22,89]

## Data Availability

The majority of the data is contained within the paper. However, in the event of a specific data point request, please feel free to reach out to the authors, who will gladly provide the required information.

## Supplementary Materials

The following supporting information can be downloaded at https://www.mdpi.com/article/10.3390/microorganisms11071728/s1. Table S1: Calculation of WoE-IV/Bins count separating animaland human studies.
