# Supplementary material for: The Role of Carbohydrate Intake on the Gut Microbiome: A Weight of Evidence Systematic Review
Source: Microorganisms. 2023 Jun 30;11(7):1728. doi: 10.3390/microorganisms11071728 (PMC10385781; doi:10.3390/microorganisms11071728)
Supplement: Supplementary file 1 [file microorganisms-11-01728-s001.zip › microorganisms-2450290-supplementary.pdf]

Supplementary table S1: Calculation of WoE-IV / Bins count separating animal and human studies

PREDICTIVE VARIABLE	RESPONSE VARIABLE	INFORMATION VALUE (IV)	BINS	REFERENCES	CORPORA
Sulfated polysaccharides	Lactobacillus decrease	1.223	1	[88]	animal
Gelatinized starch	Prevotella increase	1.610	2	[23,90]	animal
Sulfated Polysaccharides	Desulfovibrio increase	1.223	1	[18]	animal
Fungal Polysaccharides	Proteobacteria decrease	1.223	1	[114]	animal
Sulfated Polysaccharides	Turicibacter increase	1.223	1	[97]	animal
Sulfated Polysaccharides	Odoribacter increase	1.223	1	[18]	animal
Oligosaccharides	Lactobacillus increase	0.941	6	[18,24,95,104,111,113]	animal
Oligosaccharides	Enterococcus decrease	0.777	2	[24,104]	animal
Resistant starch	Firmicutes increase	0.770	1	[96]	animal
Starch	Firmicutes increase	0.620	1	[96]	animal
Carbohydrates naturally occurring in food	Bacteroides increase	0.975	2	[111,113]	animal
Insoluble fiber	Bacteroides increase	0.594	4	[90,92,97,111]	animal
Soluble fiber	Bacteroides increase	0.974	5	[90,92,97,109,112]	animal
Insoluble fiber	Firmicutes decrease	0.371	4	[99,108,110,111]	animal
Sulfated Polysaccharides	Roseburia increase	1.223	1	[97]	animal
Sulfated Polysaccharides	Enterococcus decrease	0.558	1	[97]	animal
Fructan	Faecalibacterium increase	0.347	1	[122]	human

(1) Chen, P.; Yang, S.; Hu, C.; Zhao, Z.; Liu, J.; Cheng, Y.; Wang, S.; Chen, Q.; Yu, P.; Zhang, X.; Wu, M. Sargassum Fusiforme Polysaccharide Rejuvenat Es the Small Intestine in Mice Through Altering Its Physiol Ogy and Gut Microbiota Composition. *Curr Mol Med* **2017**, *17* (5). https://doi.org/10.2174/1566524018666171205115516.
(2) Pi, Y.; Mu, C.; Gao, K.; Liu, Z.; Peng, Y.; Zhu, W. Increasing the Hindgut Carbohydrate/Protein Ratio by Cecal Infusion of Corn Starch or Casein Hydrolysate Drives Gut Microbiota-Related Bile Acid Metabolism To Stimulate Colonic Barrier Function. *mSystems* **2020**, *5* (3), 1–16. https://doi.org/10.1128/msystems.00176-20.
(3) Surono, I. S.; Venema, K. Modulation of Gut Microbiota Profile and Short-Chain Fatty Acids of Rats Fed with Taro Flour or Taro Starch. *Int J Microbiol* **2020**, *2020*. https://doi.org/10.1155/2020/8893283.
(4) Shang, Q.; Shi, J.; Song, G.; Zhang, M.; Cai, C.; Hao, J.; Li, G.; Yu, G. Structural Modulation of Gut Microbiota by Chondroitin Sulfate and Its Oligosaccharide. *Int J Biol Macromol* **2016**, *89*, 489–498. https://doi.org/10.1016/j.ijbiomac.2016.04.091.
(5) Zhong, L.; Ma, N.; Zheng, H.; Ma, G.; Zhao, L.; Hu, Q. Tuber Indicum Polysaccharide Relieves Fatigue by Regulating Gut Microbiota in Mice. *J Funct Foods* **2019**, *63* (April), 103580. https://doi.org/10.1016/j.jff.2019.103580.
(6) Zhu, Z.; Zhu, B.; Sun, Y.; Ai, C.; Wu, S.; Wang, L.; Song, S.; Liu, X. Sulfated Polysaccharide from Sea Cucumber Modulates the Gut Microbiota and Its Metabolites in Normal Mice. *Int J Biol Macromol* **2018**, *120*, 502–512. https://doi.org/10.1016/j.ijbiomac.2018.08.098.
(7) Yan, S.; Shi, R.; Li, L.; Ma, S.; Zhang, H.; Ye, J.; Wang, J.; Pan, J.; Wang, Q.; Jin, X.; Liu, X.; Liu, Z. Mannan Oligosaccharide Suppresses Lipid Accumulation and Appetite in Western-Diet-Induced Obese Mice Via Reshaping Gut Microbiome and Enhancing Short-Chain Fatty Acids Production. *Mol Nutr Food Res* **2019**, *63* (23), 1900521. https://doi.org/10.1002/mnfr.201900521.
(8) Zhang, S.; Hu, H.; He, W.; Muhammad, Z.; Wang, L. Regulatory Roles of Pectin Oligosaccharides on Immunoglobulin Production in Healthy Mice Mediated by Gut Microbiota. **2019**, *1801363*, 1–8. https://doi.org/10.1002/mnfr.201801363.
(9) Peerakietkhajorn, S.; Jeanmard, N.; Chuenpanitkit, P.; K-da, S.; Bannob, K.; Khuituan, P. Effects of Plant Oligosaccharides Derived from Dragon Fruit on Gut Microbiota in Proximal and Distal Colon of Mice. *Sains Malays* **2020**, *49* (3), 603–611. https://doi.org/10.17576/jsm-2020-4903-15.
(10) Ou, J.; Huang, J.; Song, Y.; Yao, S.; Peng, X. Feruloylated Oligosaccharides from Maize Bran Modulated the Gut Microbiota in Rats. *Plant Foods for Human Nutrition* **2016**. https://doi.org/10.1007/s11130-016-0547-4.
(11) Monteagudo-Mera, A.; Arthur, J. C.; Jobin, C.; Keku, T.; Bruno-Barcena, J. M.; Azcarate-Peril, M. A. High Purity Galacto-Oligosaccharides Enhance Specific Bifidobacterium Species and Their Metabolic Activity in the Mouse Gut Microbiome. *Benef Microbes* **2016**, *7* (2), 247–264. https://doi.org/10.3920/BM2015.0114.

(12) Kaur, A.; Chen, T.; Green, S. J.; Mutlu, E.; Martin, B. R.; Rumpagaporn, P.; Patterson, J. A.; Keshavarzian, A.; Hamaker, B. R. Physical Inaccessibility of a Resistant Starch Shifts Mouse Gut Microbiota to Butyrogenic Firmicutes. *Mol Nutr Food Res* **2019**, *63* (7), 1–8. https://doi.org/10.1002/mnfr.201801012.
(13) Chijiiwa, R.; Hosokawa, M.; Kogawa, M.; Nishikawa, Y.; Ide, K.; Sakanashi, C.; Takahashi, K.; Takeyama, H. Single-Cell Genomics of Uncultured Bacteria Reveals Dietary Fiber Responders in the Mouse Gut Microbiota. *bioRxiv* **2019**, 1–14. https://doi.org/10.1101/784801.
(14) Higashimura, Y.; Baba, Y.; Inoue, R.; Takagi, T.; Mizushima, K.; Ohnogi, H.; Honda, A.; Matsuzaki, Y.; Naito, Y. Agaro-Oligosaccharides Regulate Gut Microbiota and Adipose Tissue Accumulation in Mice. *J Nutr Sci Vitaminol (Tokyo)* **2017**, *63* (4), 269–276. https://doi.org/10.3177/jnsv.63.269.
(15) Zhou, S.; Xu, J.; Zhu, H.; Wu, J.; Xu, J.; Yan, R.; Li, X. Gut Microbiota-Involved Mechanisms in Enhancing Systemic Exposure of Ginsenosides by Coexisting Polysaccharides in Ginseng Decoction. *Nature Publishing Group* **2016**, No. September 2015, 1–13. https://doi.org/10.1038/srep22474.
(16) Chang, S.; Cui, X.; Guo, M.; Tian, Y.; Xu, W.; Huang, K.; Zhang, Y. Insoluble Dietary Fiber from Pear Pomace Can Prevent High-Fat Diet-Induced Obesity in Rats Mainly by Improving the Structure of the Gut Microbiota. *J Microbiol Biotechnol* **2017**, *27* (4), 856–867. https://doi.org/10.4014/jmb.1610.10058.
(17) Tian, G.; Wu, X.; Chen, D.; Yu, B.; He, J. Adaptation of Gut Microbiome to Different Dietary Non-Starch Polysaccharide Fractions in a Porcine Model. **2017**, 1–43. https://doi.org/10.1002/mnfr.201700012.
(18) Ferrario, C.; Statello, R.; Carnevali, L.; Mancabelli, L.; Milani, C.; Mangifesta, M.; Duranti, S.; Lugli, G. A.; Jimenez, B.; Lodge, S.; Viappiani, A.; Alessandri, G.; Asta, M. D.; Rio, D. Del; Sgoifo, A.; Sinderen, D. Van; Ventura, M.; Turroni, F. How to Feed the Mammalian Gut Microbiota : Bacterial and Metabolic Modulation by Dietary Fibers. **2017**, *8* (September), 1–11. https://doi.org/10.3389/fmicb.2017.01749.
(19) Healey, G.; Murphy, R.; Butts, C.; Brough, L.; Whelan, K.; Coad, J. Habitual Dietary Fibre Intake Influences Gut Microbiota Response to an Inulin-Type Fructan Prebiotic: A Randomised, Double-Blind, Placebo-Controlled, Cross-over, Human Intervention Study. *British Journal of Nutrition* **2018**, *119* (2), 176–189. https://doi.org/10.1017/S0007114517003440.
